# Address by J. A. Peters, M. D., before the Erie County Medical Society at Its Annual Meeting

**Published:** 1864-03

**Authors:** 


ART. II.—*Address by* J. A. PETERS, M. D., *before the Erie County Medical Society at its Annual Meeting.*

[Published by vote of the Society.]

*Mr. President and Gentlemen:*

I had hoped, as you undoubtedly did, that the orator for the occasion, (Dr. Mackay,) would be able to regale us at this time from his varied stores of knowledge and experience, but he has been unable so to do, and I am compelled to appear in his stead. Under these circumstances, quite as unpleasant to me as to you, I have tried, to the best of my ability, to perform the task set me in an acceptable manner, and I hope the attempt may be received with indulgence, if it do not merit your approbation.

It is certainly neither new nor startling to assert that there are fashions in medicine as in nearly everything else, and that, to a certain extent, they tend to re-produce themselves. As a pendulum when forced far to one side of the perpendicular, flies just as far to the opposite side, and then, passing through shorter and shorter arcs, finally settles in the perpendicular; so the mind of man, forced, either in religion, science, or politics, from that centre in which Truth lies, is inclined to fly to the opposite extreme, and it is only by constant endeavors, and unwearied labor, that it settles upon the truth. In nothing is this more clearly shown than in the various modes which have had their rise, decline, and fall in the domain of Therapeutics. Not very many years ago, physicians, armed with the lancet in one hand, and a formidable array of anti-phlogistics in the other, waged a guerrilla warfare upon nearly every form and phase of disease. Later, however, they seem to have retired from the list of active combatants, and to have become subordinates to the *vis medicatrix naturæ*, whoever or whatever that may be; and while they "sustained and supported" the patient watched the fluctuations of the combat with philosophical eye. Was this because they had learned that all diseases arise from a common morbid action which tends to lower vitality, or was it simply the recoil of the pendulum? I fear the latter; let us hope, however, that through shortening arcs we are finding the truth.

This end is to be achieved, not by empirical reasoning on the *post ergo propter* principle, from ever so well ascertained facts, but by the careful collating and comparing of clinical results, with the ascertained truths of physiological science, in order that, so far as in us lies, we may find out the reason why we give a remedy, and the mode whereby it produces its effect; and this result must be equally distant from, and indebted to, the routinism of the mere practitioner, and the theorizing of the mere physiologist. And in proportion as this is honestly and fearlessly done by every physician for himself, according to his abilities, and opportunities, just in that proportion will the tendency to fashions in the profession be done away with, and just so much the sooner will Truth be arrived at. If we permit ourselves to see through other men's eyes, hear through their ears, and think with their brains, if we do not, on the contrary endeavor to form our own ideas, though with all due humility, we are recreant to the cause of Truth in Science, a cause which we are bound, as members of a learned profession to uphold to our utmost.

Three questions must be answered in regard to every medicine before we can be considered to use it understandingly:

I.—What effect does it produce on the system?

II.—How does it produce this effect?

III.—What are its therapeutical uses?

In this paper I purpose endeavoring to answer these questions, as applied to Quinine, according to my belief.

First, then, What is the effect of Quinine on the system?

The answer, if we seek to go no farther than its classification, is ready to our hands—it is a tonic and an anti-periodic. But names are of use in science only when they convey, not when they cover up ideas, and therefore we must be sure we understand clearly what sort of action is implied by these two terms, and it is just here that we find ourselves, if we examine the authorities, afloat on a sea of conjecture and theory. But it is not necessary, in order to define these terms with sufficient accuracy to answer our present purpose, to enter very deeply into the discussion. In regard to the term "anti-periodic" no difficulty is experienced, every one understanding it to be a medicine given with a view to remove the element of periodicity in disease, but the former term "tonic," has been variously defined by different authors. Headland, (Action of Medicines, p. 159) restricts the term tonic to the bitter principles of vegetables, and maintains that they act by adding some needed element to the blood. Dr. Wood (Dispensatory) defines them to be medicines "which moderately and permanently exalt the energies of all parts of the system, without necessarily producing any apparent increase of the healthy actions." Dunglison ranks them as "medicines which have the power of exciting slowly, and by insensible degrees, the organic actions of the different systems of the animal economy —and of augmenting their strength in a durable manner," But without entering into the minutiæ of the difinition, I prefer to leave the term to that general definition which every man has, more or less clearly in his mind. We all recognize a quality termed *tone* in the system in health, and are at no loss to determine when it is absent in disease, precisely of what it consists is of minor importance in this place. Let us then define a tonic to be a medicine which has the power of restoring tone to the enfeebled system, and proceed to consider,

II.—How does Quinine produce its effects on the system?

And here we get if possible a greater variety of answers than to our former question. Says one author, it acts by adding something which is wanting to the blood; another considers that it acts on the nervous system as a stimulant, another, that it acts as a catalytic, inducing changes in the blood; and a fourth thinks it may act in all these ways. Some say that it increases the tonic, or contractile power of the muscles; others that it is a sedative to the nerves; some that it acts on the cerebro-spinal centres; others the ganglionic system, and so on, and on, and on.

It seems to me, however, that we must believe it to act in one of two modes, *i. e.*, either upon the blood, or upon the nervous system. It may act upon the blood in three ways, as a depurative, eliminating from the blood some foreign and deleterious element; as a catalytic, inducing by its presence some change in the constitution of the blood; or as a restorative, adding to the blood some needed ingredient of which it has been deprived by disease.

I cannot satisfy myself that we have any evidence beyond conjecture and assertion, to prove that Quinine has any power over the blood as a depurative, or as a catalytic—medicines which act in that way not usually being tonics, in the true sense of the word, and without further argument I think it may be assumed that, if Quinine acts on the blood at all, it does so in the way last named, *i.* e. as a restorative.

If it act on the nervous system it must produce either a stimulant or a sedative effect; both have been alleged in regard to it, both are supported by many facts, and it seems to me that both may be true. Instances are not wanting of other medicines which act as stimulants to the cerebro-spinal centres in certain doses, becoming sedatives when given in increased quantities, indeed, I am not sure that this is not generally the effect of a stimulant pushed beyond a certain point. But I shall have occasion to refer again to this question, and merely saying that in my judgment the action of Quinine is either produced in the blood as a restorative, or on the nervous system as a stimulant, I shall proceed to enquire which of these theories is correct.

And first, let us glance at the phenomena produced by its administration, so that we may judge whether the first or the second of these hypotheses will best account for them. I do not refer to the ultimate or general effect on the system, for which we give the remedy, but to the immediate and transient effects which mark the mode and direction of its action. These are, a sense of warmth at the epigastrium, and a general glow which gradually diffuses itself over the whole system. Under certain circumstances the heart's action is increased, and the brain stimulated almost to delirium; a sense of dizziness is experienced, a ringing in the ears as if of a chime of bells, and sometimes a temporary deafness. Again, under favorable circumstances a sedative effect is produced, and sleep follows its action. *All* these phenomona may not be observed in *every* case, but more or less of them take place whenever quinine is administered either in health or disease.

Now these effects have been said to mark the action of Quinine as an irritant poison, by those who maintain that it acts only on the blood, but I cannot find any reason for ranking it among irritant poisons, except the fact that in certain states of the stomach it produces, or increases, irritation of that organ, a property which exists in many other articles certainly not poisons. On the other hand, they seem clearly to me to indicate that a stimulant effect has been produced on the nervous system; and I have known instances where Quinine has been taken for its stimulant effect in health. The lamented Dr. Wilcox, late a member of this Society, was accustomed so to take it when undergoing severe exposure to wet or cold, declaring that it "keyed him up" and made him "feel warm."

If Quinine act only as a restorative to the blood, then its action should be slow and almost imperceptible, and no direct effect on the nervous system, and no perceptible effect on a healthy man should be produced. We give iron in anæmia, because we know the blood to be deficient in that metal, and we hope to restore it, and we all know at how slow a rate the process of restoration goes on. While this theory might not be incredible if confined to the action of Quinine as a tonic, it hardly seems reasonable when we are asked to believe that it may be absorbed with such great rapidity as it must be in cases of periodical disease, so as to limit an ague, for instance to a single paroxysm. Nor have we any proof beyond conjecture that quinine can replace any ingredient of the blood. We are told that in debility there is a deficiency of taurine in the blood, Quinine resembles taurine in its ultimate chemical composition; Quinine cures debility, hence Quinine replaces taurine in the blood. It is also conjectured that the same pathological condition exists in all the diseases wherein Quinine is applicable, hence that is the way in which the remedy always acts. To me I confess, notwithstanding the high authority by which it is supported, this reasoning looks very much like a *non sequitur*. It should not be forgotten that identity of chemical composition, in substances of organic origin, is very far from implying identity of *organic* composition, or of physiological use. Nor does it seem likely to be true that any of the ingredients of the blood can be replaced by other substances *resembling* them.

Quinine is given with good effect in all cases of debility, and in all diseases which depend for their cause on malaria. Now we must believe that in all these diseases we have the same pathological condition, which condition consists in a certain deficiency of the blood, to be supplied by Quinine, or else the remedy must act through some other channel.

An attempt has indeed been made to reconcile the conflicting theories by suggesting that both may be true, that Quinine may act at one time as a restorative to the blood, and at another as a stimulant to the nervous system, but notwithstanding this idea has been supported by high authority, I cannot bring myself to believe that a medicine ever acts at different times, in modes inconsistent with one another. Examples are numerous of different *effects* being obtained by giving the same medicine in different doses, but that is very far from implying a radical difference in its mode of action at different times. I doubt whether we have any sufficient proof that, in all these diseases, the same condition of the blood exists, nor do I allege that a condition of the nervous system, common to them all, is absolutely proven; but it seems quite as reasonable to suppose that the same *general* condition of the nervous system is to be found, in a greater or less degree, in them all, and is met by the same remedy acting in a uniform and consistent manner. Quinine can be readily supposed to so stimulate the nervous system in diseases of debility, as to increase the physiological activity of the various organs of the body, causing more nourishment to be assimilated, and so curing the debility. And this view certainly tallies more nearly with its effects in diseases of malarial origin. For in all these diseases we certainly have a powerful impression produced on the nervous system, and it is a curious and interesting study to watch, in a large body of men exposed to the same malarious influences, the various grades of affections induced, from congestive fevers to mild neuralgis. Nay, we may often witness most, or all of these, in the same individual. A man who is attacked, in a malarial locality, with a severe intermittent, will very often find it become, on removing to a healthier atmosphere, a periodic neuralgia. Whatever may be the pathological effect of malaria in the blood, I believe that it produces a powerful depressing effect on the nervous system, (whether the ganglionic or the cerebro-spinal, I shall not stop to inquire,) and that Quinine cures the disease by producing a counter-impression, which enables the system to resist, and finally to triumph over the morbid cause. Very often an attack of intermittent consists of but one paroxysm, the prompt administration of the alkaloid preventing its re-appearance, and I cannot believe that a deficiency in the blood of any one of its elements can be supplied in so short a time, while it is undoubtedly true that an overwhelming impression on the nervous system, whether for good or ill, may be produced very soon.

The conclusion, then, to which I have been driven, and which I wish, with all due humility, to present, is that Quinine acts on the system by producing a stimulant impression on the nerves, and that its effect on the blood is only indirect. Let it be understood, however, that I use the term stimulant only to indicate the direction and mode of its action, and not to measure or limit its rapidity or results.

III. What are the therapeutical uses of Quinine?

Whatever views may be held by the practitioner regarding its mode of action, the great practical question becomes: In what diseases is it applicable? I have adverted to the fact of quinine being a tonic and an anti-periodic, and it justly stands at the head of these classes of remedies, yet I believe it has come to be one of the fashions of the day, and is often given —perhaps with good effect—without any very clearly defined reason for so doing. To a great extent I am convinced it has usurped the place formerly held by mercury as a *pis aller.* Said an old physician, "all of the olden time," to his pupil, "When you don't know what to give, give calomel, and you can't be far out of the way." Have none said, at least to themselves, "When at a nonplus, give quinine, and you can't do any hurt?" Now this is something a shade worse than empiricism, for it is founded on no reason, theory, or experience. It is the resort of the lazy man, the man who steals all his ideas, and has not always the wit to steal them correctly.

I have collected a list of diseases in which Quinine has been given, and though I do not suppose it to be full, still it shows how much faith has been placed in the remedy in conditions apparently the most opposite. It has been given in all diseases of debility, in intermittent, remittent and continued fevers, in scarlatina, measles and small-pox, in carbuncle, gangrene and erysipelas, in scrofula, dropsy, passive hæmorrhages, dyspepsia, obstinate cutaneous affections, amenorrhœa, chorea, hysteria, in acute rheumatism, neuralgia, hemicrania, in epilepsy, diarrhœa, dysentery, and has been highly recommended as an anthelmintic.

Quinine may be given in tonic doses whenever, in the course of any disease, there is a loss of tone in the nervous system, or a deficiency of digestive power, provided always that there be no such vitiated condition of the stomach as to render it worse than useless. It is most useful—indeed, I am inclined to say *only* useful in tonic doses—when there seems to be chiefly a lack of sufficient nervous energy to carry on the processes of assimilation, and nutrition, when the stomach can tolerate food, but the stimulus of healthy appetite is lacking. Then Quinine seems to rapidly and steadily bring about a normal condition; under its influence more food is taken into the stomach, and more of it assimilated, and gradually and steadily, strength and health are restored to the wasted frame. But when, in the course of febrile or inflammatory diseases, there is a high state of febrile action, and the dry, brown tongue indicates the irritation going on in the stomach and in the whole system. I believe quinine, *as a tonic*, only exacerbates the morbid action already existing. Weakness there certainly may be, and need for alimentation, which need must be met by *food*, as beef essence, not by Quinine. Let the tonic be withheld until, by the use of alteratives, or by the spontaneous recedence of the disease, the morbid action has disappeared and our patient only needs to be raised from his enfeebled condition. There is small use in trying to bind up the wounds of a combatant while he is still fighting, though after the fray our dressings may be grateful and necessary. I have often seen this mistake made, (I am not at all sure I did not make it myself before I learned better) in treating a certain class of fevers often found in camp, which are not malarial in their origin, but which, arising from the exhalations incident to such places, assume a typhoid character, though not true typhoid fever. These fevers have been called typhoid, and treated with Quinine as a matter of course, very much to the cost of the government and the injury of the patient, (*vide* a paper by Dr. S. B. Hunt in the Buff. Med. and Surg. Journal, vol. II., p. 202). It is not necessary to enumerate the diseases in which quinine is useful as a tonic; they must necessarily be left to the discretion of the practitioner.

In all diseases of malarial origin, whether fevers or neuralgias, Quinine is our first resort, and most commonly our sheet anchor. Nay, when we suspect any disease under our care to be *modified* by malarial influences, we will find Quinine, in anti-periodic or anti-malarial doses to be a useful adjuvant to our other treatment. In all such cases, whether of intermittent, remittent, or continued fever, I believe the most rational, as well as the most humane plan, is to give the remedy in such large doses as to cut the disease short in the briefest possible time. The opposite course only prolongs the sufferings of our patient, and exposes his system to be the more enfeebled by the disease. Regard must be had to the locality as well as to the temperament of the patient. The more intense the character of the malaria, the larger must be the dose. What would be a large dose here in Western New York, would be of no avail on the banks of the Potomac. My own plan, in treating intermittent in the South, has been to give full doses, (5 to 10 grains) every four to six hours, commencing immediately after a paroxysm, and continuing until about half an hour before the next paroxysm should begin, when two or three doses were given at once in some carminative such as Fluid Ext. Zingiber, or Pulv. Capsici, and the patient directed to go to bed. When a paroxysm has been prevented, the size of the dose should be gradually decreased until none is taken, when the disease will usually be cured. Of course I need not allude to the many exceptions or variations; I only give this as the plan *generally* most successful in my hands. Whether or not any preparatory treatment is necessary, is a question very often discussed and with no little warmth, but I believe it to be more a question of climate than of accuracy or veracity. I believe those who practice in notoriously malarious localities, usually deem some preparation by cathartics or alteratives necessary, while those who practice in more favored climates take the other ground. Both seem to me right in their respective fields of action. I certainly am in favor of "preparatory treatment" as a rule among the swamps of the South, and do not doubt it to be necessary in similar locations West. Here, I presume it may be dispensed with.

In remittent or continued fevers, as well as in other diseases of a distinctly malarial character, I believe the same theory of large doses holds good. It should be always remembered that a small dose of Quinine, except when given strictly as a tonic, is a stimulant, and in many cases an excitant, while in full doses it may become a sedative. In the one case, by a succession of small doses, we excite and irritate our patient, and do not affect the disease, while in the other, by a full dose, we calm and soothe the patient, and impress, perhaps cure, the disease.

This is seen in the treatment of children. perhaps better than in the case of adults, even when we are not combating the influence of malaria, as for instance when Quinine is given in scarlatina. This medicine has been highly recommended in acute rhumatism and other cases of acute febrile action not dependent for their origin upon malaria, but as my experience in its use in such cases has been limited I shall not have much to say about it. As, however, we do not need or desire in such cases a tonic, still less an irritant effect, it would seem that it should be given in full, or sedative doses.

I have used the terms large and small doses, but I wish to be always understood as using them relatively. Ten grains in certain cases, would be a much larger dose than would thirty under other circumstances.

Nor should it be forgotten that there is sometimes a certain amount of danger in the administration of Quinine, particularly when it is given in inflammatory or febrile diseases, of non-malarial origin. This danger arises from its effect upon the brain, especially when given in full doses. Given during the hot stage of intermittent, it aggravates the febrile re-action, often producing delirium, yet in congestive fevers, of a malarious character, I believe it should not be withheld, since we must depend upon its agency to remove or counteract the morbid cause. If there be local congestion, especially of the brain, it must be met by appropriate treatment, but Quinine must be relied on to effect the cure. The stage of collapse occurring in these cases needs large doses of the alkaloid, and at once. An overwhelming, depressing effect has been produced on the nervous system, and must be counteracted by the most prompt and efficient means.

To sum up—Quinine acts on the nervous system as a stimulant; in larger doses, a sedative effect is produced, which is not inconsistent with, but follows legitimately from its stimulant action; owing to these qualities it is valuable as a tonic and as an anti-periodic, or anti malarial, and also (it is said) as a *febrifuge;* notwithstanding under the counteracting influence of a depressing agent, enormous doses will be borne, are absolutely demanded; some caution is needed in its administration.

I have given my own views upon this important subject, but claim for them no more originality than every man is entitled to claim for those ideas which have been taught him by reflection upon the facts observed in his own experience, and the facts and theories promulgated by others. As to their truth, every one must judge for himself, they being put forth with that sort of mental reservation, to which a certain distinguished Professor says every physician is entitled in giving an opinion; so that, if I hereafter become convinced that they are untrue, I shall claim the privilege of contradicting them, without rendering myself liable to the reproach of inconsistency.

